# Corrections

**Published:** 1891-02

**Authors:** 


Corrections.—In Dr. Baylies' article, page 515, November No., 9th line from top, insert "*in*" before "common." December number, page 551, twelfth line from the bottom after the words, *In pregnancy* insert the words *under Phos.*

